# DHA Effects in Brain Development and Function

**DOI:** 10.3390/nu8010006

**Published:** 2016-01-04

**Authors:** Lotte Lauritzen, Paolo Brambilla, Alessandra Mazzocchi, Laurine B. S. Harsløf, Valentina Ciappolino, Carlo Agostoni

**Affiliations:** 1 Department of Nutrition Exercise and Sports, University of Copenhagen, Rolighedsvej 26, 1958 Frederiksberg C, Denmark; laurinebs@gmail.com; 2 Psychiatric Clinic, Department of Neurosciences and Mental Health, Fondazione IRCCS Ospedale Cà Granda-Ospedale Maggiore Policlinico, University of Milan, 20121 Milan, Italy; paolo.brambilla1@unimi.it (P.B.); valentina.ciappolino@libero.it (V.C.); 3 Department of Psychiatry and Behavioural Neurosciences, University of Texas at Houston, 2800 South Macgregor Way, Houston, TX 77021, USA; 4 Pediatric Clinic, Fondazione IRCCS Ospedale Cà Granda-Ospedale Maggiore Policlinico, Department of Clinical Sciences and Community Health, University of Milan, 20121 Milan, Italy; alessandra.mazzocchi1@gmail.com (A.M.); carlo.agostoni@unimi.it (C.A.)

**Keywords:** docosahexaenoic acid, brain development, desaturases, psychiatric disorders

## Abstract

Docosahexaenoic acid (DHA) is a structural constituent of membranes specifically in the central nervous system. Its accumulation in the fetal brain takes place mainly during the last trimester of pregnancy and continues at very high rates up to the end of the second year of life. Since the endogenous formation of DHA seems to be relatively low, DHA intake may contribute to optimal conditions for brain development. We performed a narrative review on research on the associations between DHA levels and brain development and function throughout the lifespan. Data from cell and animal studies justify the indication of DHA in relation to brain function for neuronal cell growth and differentiation as well as in relation to neuronal signaling. Most data from human studies concern the contribution of DHA to optimal visual acuity development. Accumulating data indicate that DHA may have effects on the brain in infancy, and recent studies indicate that the effect of DHA may depend on gender and genotype of genes involved in the endogenous synthesis of DHA. While DHA levels may affect early development, potential effects are also increasingly recognized during childhood and adult life, suggesting a role of DHA in cognitive decline and in relation to major psychiatric disorders.

## 1. Introduction

Long chain polyunsaturated fatty acid (LC-PUFA), including docosahexaenoic acid (DHA) and arachidonic acid (AA), are incorporated into membrane phospholipids and, apart from their structural role in these membranes, they also act as precursors of autocoid signaling molecules (e.g., docosanoids) and as potent activators of a number of gene transcription factors (e.g., peroxisome proliferator activated receptors). The essentiality of n-3 LC-PUFA is generally mainly contributed to the incorporation of DHA in uniquely high levels in the central nervous system—although DHA is incorporated in most other tissues where it may also have important functional effects.

Overall, membrane PUFA composition (the principal components of which are linoleic acid (LA), AA and DHA) seems to be more responsive to DHA in the diet than to intake of LA and AA [1]. Animal studies have demonstrated that an increase in dietary α-linolenic acid (ALA) is almost completely reflected in membrane n-3/n-6 PUFA-ratios at LA/ALA intakes of <10, whereas the dietary balance between ALA and LA has little influence at higher ALA intakes, and a similar biphasic response is also seen in diets that contain LC-PUFA [2]. These results show a high sensitivity of tissue membranes to dietary variations in the PUFA-supply within the normal range, strongly favoring incorporation of n-3 LC-PUFA over LA and AA. In the case of a dietary deficiency of n-3 PUFA, there is a trend for DHA to be replaced with the nearest n-6 PUFA equivalents, whereas few changes are seen for the reciprocal lack of dietary of n-6 PUFA [3,4]. Thus, n-3 PUFA seem to be the main determinant of membrane PUFA composition and unsaturation. Membrane DHA incorporation in different tissues, e.g., erythrocytes (RBC), has been shown to depend on diet, mainly fish intake and in infants also breastfeeding, but is also to some extent supported by DHA formed endogenously by desaturation and elongation of ALA. This conversion is limited by the delta-6 desaturase enzymatic step, which generally has a low efficiency, but the rate conversion has been shown to be affected by genetic setup in the fatty acid desaturase (*FADS*) gene cluster and to vary depending on age and circulating levels of sex hormones.

The present paper will give an update of the current literature and try to answer the following questions: (1) Does the high rate of DHA accumulation in the brain have any functional importance? (2) If yes, is the endogenous synthesis of DHA high enough to support optimal functional levels of DHA in the brain? Finally, we will also address whether DHA may contribute to normal brain functioning later in life.

## 2. Brain DHA Accumulation during Development Depending on Diet

The accumulation of DHA in the brain takes place during the brain growth spurt in the intrauterine and neonatal period up to two years of age and the high levels of DHA in the brain are maintained throughout life [5]. Due to the lack of *de novo* PUFA synthesis, the rate of membrane DHA incorporation in early life—in the brain as well as in other tissues—depends on maternal transfer, dietary supply (*i.e.*, breastfeeding) and endogenous LC-PUFA production. The DHA accumulation in the brain during the third trimester of pregnancy is substantially higher (in % of fatty acids (FA%)) than the overall body deposition rates, whereas brain incorporation of AA is more in line with that which occurs in other tissues [6]. Fetal LC-PUFAs accumulation occurs mainly during the last trimester, in which weight increase becomes more rapid and growth is accompanied by a deposition of fat tissue, which begins around the 30th week of gestation [7]. Fetal fat tissues contain relatively low levels of DHA and AA [8,9] compared to the large relative amounts of LC-PUFAs that are deposited in the brain [8,10]. However, the absolute amount of DHA in fetal adipose tissue exceeds that in the brain [7]. Based on *post-mortem* studies it has been calculated that whole-body DHA accretion during the third trimester amounts to around 50 mg/day while the accretion of AA is approximately twice as high (100 mg/day) [8]. It has been estimated that this fetal LC-PUFA accumulation is supported by a supply of approximately 50 mg/(kg × day) of n-3 LC-PUFA and 400 mg/(kg × day) of n-6 LC-PUFA [6].

The intrauterine PUFA supply occurs via transfer of non-esterified PUFA mainly derived from the maternal circulation across the placenta [10,11]. The overall fat concentration in maternal plasma increases throughout pregnancy [7], and placental fat transport is driven by a concentration gradient as the fetus has substantially lower fat concentrations [12], including the concentration of DHA and AA [7,13]. The relative proportion of DHA and AA is, however, consistently higher in circulating lipids of the neonate [14], whereas the concentrations of LA and ALA differ much less from that in the maternal blood [15,16], indicating a preferential transfer of LC-PUFA. The exact mechanisms involved in placental PUFA transfer remain unclear, but is generally considered to involve proteins with some specificity for LC-PUFA, especially DHA, over PUFAs with shorter chain length [17,18,19]. Additionally, DHA has been shown to be incorporated into triacylglycerol in human placental cells, whereas AA is primarily esterified in phospholipids [20,21], and this differential esterification may contribute to the preferential transport of DHA and accumulation of AA in the placenta itself. AA has been shown to be taken up from the maternal blood by the placenta at higher rates than DHA, while DHA accumulates in the fetal blood stream at a three-fold higher rate than AA [20]. Although other interpretations are plausible, the specificity for placental transfer of DHA over AA could be interpreted as a specific retention of AA on the maternal side possibly for prostaglandin production in relation to the initiation of delivery. Maternal dietary n-3 LC-PUFA has a slight gestation prolonging effect, which may be explained by a dampening of the AA-derived eicosanoid response [22], which results in an increase birth weight and intrauterine LC-PUFA accretion. In infants born preterm the progressive accumulation of LC-PUFA in fetal tissues is truncated at the end of pregnancy and accumulation is also strongly limited in growth-retarded fetuses [23].

Post-natal accumulation of LC-PUFA in infant tissues is supported by maternal transfer of PUFA through breastmilk, and blood levels of LC-PUFA in breast-fed infants remain higher than maternal levels for some time postnatally [24,25]. In neonate baboons, dietary DHA has been shown to consistently support greater brain DHA incorporation and maintenance of cortex DHA concentration, while brain AA is unaffected by dietary supply and decreases with age [26]. Moreover, brain autopsies from human infants have shown an around 25% higher mean FA% of DHA in cortical phospholipid of breast-fed (9.7%) compared to age-matched formula-fed infants (7.6%) [27]. The overall percentage of LC-PUFA was maintained in formula-fed infants by a compensatory increase in the incorporation of n-6 LC-PUFA, which however was incomplete in formula-fed preterm infants with the lowest concentration of cortical DHA, where an increase in the n-9 series PUFA was also detected [27]. A second autopsy study also showed an increase in cortex DHA with age in breast-fed but not in formula-fed infants, whereas the percentage of AA in the brain increased with age irrespective of diet [28] just as in the infant baboons. Similarly, the RBC DHA content of breast-fed infants has been found to be higher than that of formula-fed infants [28]. Breastmilk has been shown to be a main contributor to the DHA content in infant RBC [29], and infant RBC DHA has been shown to be associated with maternal n-3 LC-PUFA intake and RBC DHA status during lactation [30]. RBC DHA decrease after infancy as complementary feeding usually supplies less DHA [31]. The intake of n-3 LC-PUFA has been shown to be low in a number of studies in children [32] and European children have been shown to have whole blood n-3 LC-PUFA levels consistently below 2.5 FA% between 3 and 8 years of age [33].

The phenomenon of increasing LC-PUFA in fetal and infant blood and tissues relative to that of their mother has been described as “bio-magnification” [34], but could also be interpreted as a natural consequence of a dual liver system *i.e.*, the combined PUFA metabolism and conversion of LA and ALA to AA and DHA in both the mother and the fetus/infant. Both term and preterm infants have been shown to convert stable isotope labeled LA and ALA to AA and DHA, respectively [35,36,37], and the synthesis has been shown to decrease with post-conceptional age [38]. The desaturase capacity has been estimated to be in the order of 40 mg/(kg × day) of AA and 13 mg/(kg × day) of DHA in neonates born in the 32nd week of gestation, but to decrease to around 14 and 3 mg/(kg × day) at 1 month past expected term [39]. This synthetic rate may still provide a substantial contribution to fulfill infant needs, which, based on maintenance of plasma DHA homeostasis, have been estimated to be around 5 mg/(kg × day) of DHA [40]. However, this does not exclude that exogenous sources of DHA are needed in the diet to fulfill the requirements of the growing infant.

## 3. Effects of FADS Polymorphisms on LC-PUFA Levels

Overall, data suggest that n-6 PUFAs in breastmilk, plasma and RBC membranes across all ages are more affected by single nucleotide polymorphisms (SNPs) in the *FADS* gene cluster than n-3 PUFAs, typically with an increase in LA and a decrease in AA levels in minor allele carriers [29,41,42,43,44,45]. Minor allele homozygotes of various *FADS* SNPs have also been found to have lower blood (RBC and plasma) levels of AA and higher levels of LA and ALA during pregnancy [41,42]. *FADS* polymorphisms have been estimated to explain as much as 29% of the variation in serum AA contents in adults, in whom serum DHA concentrations are determined primarily by the dietary supply of preformed DHA [43]. Colostrum AA and DHA levels have been found to be decreased in minor allele carriers of a number of *FADS* SNPs [46], but studies in mature breastmilk have shown that the concentration of AA is influenced to a larger extent than that of DHA [41,47,48]. Findings in plasma from both mothers and neonates have shown strong inverse associations between the minor allele for two *FADS* SNPs and the concentrations of DHA and eicosapentaenoic acid (EPA) as well as AA in the newborn infants, thus confirming that synthesis of DHA provides a relevant contribution to status [49]. Curiously, a study of 2000 cord blood samples found that minor allele *FADS* SNPs in the mother gave rise to increased levels of n-6 PUFA before the delta-5 desaturation step (LA and di-homo-γ-linoleic acid), whereas minor allele SNPs in the child resulted in decreased levels of AA and other n-6 LC-PUFA beyond this point in the metabolic pathway [50]. More data on the biochemical effects of *FADS* polymorphisms are needed to derive a biologically plausible interpretation of their potential functional effects. Furthermore, both AA and DHA needs to be considered together since apart from the main determinants of their levels, either endogenous or exogenous, their balance may be critical for the functional outcomes in infancy and beyond.

We have recently found that some *FADS* polymorphisms may substantially contribute to RBC DHA levels in late infancy (to the same extent as breastfeeding) [29]. Some SNP minor alleles (rs1535 and rs3834458) were even found to dose-dependently up-regulate DHA status [29], whereas minor alleles of all the investigated SNPs lowered AA in a consistent way [51]. Interestingly, identical analyses did not reveal any effect of these SNPs on RBC DHA at 3 years of age [29], which could be explained by increased residual variation in the model due to a more diverse fish intake or could be interpreted as a decline in the endogenous DHA biosynthesis, consistent with other findings [39]. Furthermore, a longitudinal study of serum phospholipid fatty acid composition at 2 and 6 years of age in 331 children found higher tracking in n-3 LC-PUFA levels in children who were major allele carriers [52]. Instead tracking of n-6 LC-PUFA was lower in major allele homozygotes of various *FADS* SNPs compared to tracking in carriers of at least one minor allele [52]. More longitudinal outcome data may suggest plausible biological interpretations. However, although DHA may mainly be determined by variation in intake, mainly of preformed DHA, the genetic patterns also appear to be of relevance for tissue DHA levels in the perinatal phases, although probably less later in life as the rate of endogenous synthesis declines, thus increasing the importance of exogenous DHA.

## 4. Dietary DHA and Postnatal Development

The majority of the randomized controlled trials investigating the effect of dietary LC-PUFA supplementation in term infants have added both DHA and AA, and only few have investigated the effect of varying DHA intakes at a constant intake of AA. With respect to the functional effects of LC-PUFA supplementation in infancy, the most accepted developmental effect is an increased rate of visual acuity development [53]. This effect seems to be explained solely by DHA, as a meta-regression analysis found that variability in the effects on visual acuity between studies was explained by the dose of DHA [54]. However, little is known regarding the persistency of this effect on vision and the potential effects that this early visual deficit may have on cognitive development.

Overall, meta-analyses of the randomized controlled trials that have investigated the effect of LC-PUFA supplementation on neurodevelopmental outcomes throughout the first two years of life have not shown any clear benefit of LC-PUFA addition to infant formula on development of term or preterm infants [55,56,57]. However, a meta-analysis that combined all LC-PUFA formula supplementation trials in both term and preterm infants found a trend for an effect on the Bayley scale Mental Developmental Index at around 12 months of age, which were not affected by the maturity of the infant at birth [57]. This meta-analysis did not find any effect of LC-PUFA dose, although there was a trend towards an effect of the DHA dose, but no such trend for AA [57]. The studies that have supplemented the infants with DHA indirectly via n-3 LC-PUFA supplementation of their pregnant or lactating mothers, generally provide a more clean way to study effects of the early DHA supply as this has little effect on the AA supply to the infant. A meta-analysis of randomized trials that supplemented lactating mothers with n-3 LC-PUFA showed that infants of supplemented mothers had larger heads at 2 years of age [58]. Furthermore, the meta-analyses looking at the developmental effects of maternal n-3 LC-PUFA supplements in pregnancy and lactation have suggested some effects on neurodevelopment based on a few studies [58,59]. However, at the current stage, this does not provide any definite proof that an increase in the early DHA supply improves the mental development of infants.

So far, few studies have shown that the effect of perinatal n-3 LC-PUFA supplementation may be affected by the gender of the child. In two large investigations, the DINO and DOMInO trials [60,61], an increased early DHA supply was associated with different effects on cognitive outcomes in girls and boys. A gender-treatment interaction on cognitive outcomes was also observed in a small Danish trial of maternal fish oil supplementation during lactation [62], although no clear effects were observed when the children were followed up at 7 years of age [63]. The different effects of increased DHA supply on various outcomes in girls and boys all appear to counteract the normally observed gender differences in behavior. It is not clear if these effects should be interpreted as beneficial in one gender and adverse in the other or if it is due to some other effect of DHA that diminish the cultural gender differences which we have come to perceive as normal biological differences. Interestingly, in the Danish maternal fish oil supplementation trial treatment-gender interactions were found also on blood pressure at 7 years of age [64]. Blood pressure is not normally defined as cognitive outcome, but is nevertheless affected by the central nervous system in response to anxiety. As was the case with cognitive outcomes, boys and girls in the fish oil group were found to have comparable diastolic and mean arterial blood pressures, whereas girls had higher blood pressures than boys in the control group [64]. The intervention was also found to level out gender differences on energy intake and physical activity at 7 years of age [64]. Accordingly, these results indicate that early DHA intake could also have long-term health consequences, which might be mediated effects in the brain and lifestyle choices.

Many of the available studies on the effects of maternal or, more commonly, infant n-3 LC-PUFA supplementations on neurodevelopmental outcome during infancy have several limitations, which become more and more evident as our knowledge on the physiology of LC-PUFA, and DHA in particular, progresses. The vast majority of the studies, whether on cognition of other functional outcomes or if they provide the supplement during pregnancy, lactation or to the infant in various types of formula, show a great heterogeneity with respect to LC-PUFA sources, doses of DHA (and AA) and durations of interventions. It should be noted that the methodologies for primary outcome assessment as well as age of effect examination differed between trials, and the effects in the first few years of life and potential long-term effects may be quite different. For outcomes such as neurological and cognitive development, there may be a necessity to use different tests at different ages to accommodate changes in age and maturity level. However, many trials have investigated effects on numerous outcome measures, which are often internally inconsistent, or show no apparent pattern over time. In addition, studies often have low power in terms of the number of participants and sometimes also high rates of dropouts as well as lack of intention-to-treat analysis and a sufficient description of allocation concealment. Although baseline demographic characteristics are constantly reported, often baseline n-3 PUFA intake or status is not included in the characterization. This omission is critical for the interpretation, since baseline n-3 PUFA status will likely affects the response to changes in n-3 PUFA intake—both with respect to acute and persistent functional effect. Finally, as mentioned above, the effects of early n-3 LC-PUFA supply may vary in boys and girls, and this is not taken into account in the older studies. The emerging knowledge indicates that it is critical to take these aspects into account and that the variation in these aspects complicate attempts to combine data in meta-analyses to achieve conclusions with respect to the functional consequences of the addition of LC-PUFA, even beyond the single, specific effects of DHA.

## 5. Effects of FADS Polymorphisms on Cognition and Neurobehavioral Outcomes

Current knowledge about the functional effects of *FADS* polymorphism is limited and although the most clear effect on PUFA metabolism as mentioned is a decrease in AA production, functional associations with *FADS* genotype cannot be interpreted as a consequence of a reduction in AA. The influence of *FADS* polymorphisms on LC-PUFA status—and specifically the observed variations between specific SNPs and specific LC-PUFA over time—introduces new variables to be considered in the evaluation of the effects of *FADS* genotype on development and health of young children.

**Figure 1:**
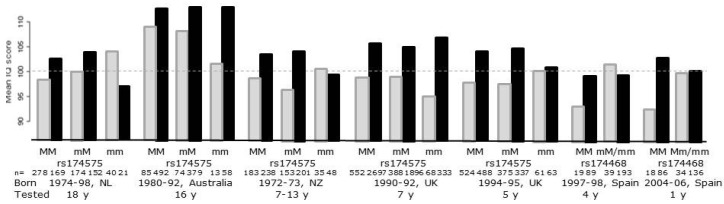
Results from studies examining the potential modifying effect of single nucleotide polymorphisms in the fatty acid desaturase gene cluster on the effect of breastfeeding on IQ-like neurodevelopmental outcomes in children. The figure is based on data from [46,65,66,67,68] and gives the average IQ in the SNP×feeding groups (breast-fed in black and formula-fed in light gray). The grey stippled line is the reference line for mean normal IQ.

Several studies have showed that infant *FADS* genotype, examined by use of different individual SNPs, modifies the effect of breastfeeding on IQ-like neurodevelopmental outcomes in childhood [46,65,66], while other studies did not find any significant interaction [67,68] (Figure 1).

As expected, based on the observed differences between breast-fed and formula-fed infants, all the studies have higher scores in breast-fed compared to formula-fed major allele carriers, but with no apparent differences between homozygotes and heterozygotes, which might be expected based on the additive effects of number of major alleles that is expected according to the observed effects on LC-PUFA. Thus, an interaction is dependent on a different pattern among the minor allele homozygotes (or the minor allele carriers in the Spanish study in which these were pooled with the heterozygotes). In the studies that found an interaction this is based on an equal “IQ” in breast-fed and formula-fed in the two cohorts in the Caspi study [66] and the two Spanish cohorts [46], whereas the largest of the studies found an even bigger difference between breast-fed and formula-fed among the minor allele homozygotes [65]. However, in all of the studies there were only few formula-fed minor allele carriers, and thus the largest variation in this group was likely skewed because of the scores of few children were at a high risk of chance effects. The studies differ with respect to breastfeeding frequency as well as the definition of breastfeeding, which in the large UK study was defined as >1 months [65] and ever having been breast-fed in the Dutch study [67], but was not clearly defined in other studies [46,66]. The Australian study tried to examine the effect of breastfeeding duration (not apparent in the figure in which we have pooled all the breast-fed groups), but they did not have the power to judge this due to a lack of a statistical (although visual indicated) dose-response between duration of breastfeeding and IQ [68]. The Dutch study found that the effect appeared to vary—although not significantly—between different cognitive functions and testing ages [67]. Furthermore, given the variation in the year of birth of the subjects in the studies, it is also reasonable to assume that there could have been differences in the PUFA composition of the formulas and presumably also in the maternal fish intake, and lifestyle in general, and thus in the DHA content of the breastmilk of the study populations. Little is known regarding interactions between the *FADS* polymorphisms and intake of AA and DHA from breastmilk and infant formula or the dietary ratio between the precursors, LA and ALA, but it is reasonable to suspect that this might have an influence on the functional response. Due to the increasing availability of micro-invasive methods for determination of blood fatty acid status, future Mendelian randomization studies should now be able to study effects of these potential sources of heterogeneity.

Additionally, the studies on interactions between breastfeeding and IQ used different *FADS* SNPs (mainly rs174575, rs1535 and rs174468), but, as indicated by the aforementioned study from Harsløf and coworkers, they may not all down-regulate the endogenous DHA synthesis in the infants [29]. This could contribute to the observed variable associations, and it is important to consider potential disequilibrium with other SNPs in the interpretation of the results from the *FADS* SNP studies. Interestingly, Steer *et al.* reported opposing effects of rs174574 and rs3834458 in the modulation of the association between breastfeeding and IQ [69]. Opposing effects of rs1535 and rs174448 have also been observed in problem solving and communication skills assessed by the Ages and Stages Questionnaire (ASQ) in a recent study among 3 year-olds [51]. None of the *FADS* SNP-breastfeeding interaction studies have considered whether the effect might differ between boys and girls. As in the DINO trials [60], the Mendelian randomization of *FADS* SNPs *versus* 3-year ASQ outcomes found that the effect of DHA increasing SNPs appeared to be negative in girls and positive in boys [51]. Due to the previously mentioned opposing effects of these SNPs on DHA in early life and the lack of opposing effects on AA plus the lack of association between *FADS* SNPs and DHA status at 3 years of age, these results might be interpreted as proof of a *programming effect* specifically of early DHA dietary intakes. However, the lack of effect of the *FADS* SNPs on DHA status later in life could be due to a blurring effect of a more diverse dietary intake of DHA from fish. Therefore, it is not possible to rule out that DHA supply contrary to the current hypothesis of an early window of vulnerability might have an effect on brain function at all stages of life.

## 6. Neurobehavioural Outcomes in Older Children

Brain DHA accretion continues into childhood, and although the accretion rate declines, the incorporation of DHA is still high at least during the preschool years. Once high levels of DHA are achieved in the brain these are maintained during later life, and this presumably also depends on an optimal dietary supply, as dietary intake of DHA from fish in adults has been shown to be the dominant determinant of DHA levels in various lipid pools [70]. However, to our knowledge no studies have examined the dietary requirements in order to achieve optimal brain DHA maintenance. Few studies have investigated the effect of *FADS* SNPs or n-3 LC-PUFA supplementation on cognitive development, emotions and behavior in toddlers and later in childhood or even in healthy adults.

A single study pooling data from three trials that randomized to LC-PUFA formulas immediately after birth or after breastfeeding for 6 weeks or 4–6 months, respectively and continued supplementation throughout the first year of life, found significant beneficial effects on problem solving at 9 months of age only in the two studies that started intervention early [71]. However, one study that examined the effects of DHA-enriched baby food also found an apparent improvement of cognitive outcomes [72]. Furthermore, a trial that provided a teaspoonful of cod liver oil (free of vitamin A and D) from 9 to 12 month of age found an increase in voluntary attention in a free play test after the intervention, especially in boys, compared with un-supplemented children [73].

Results from studies in schoolchildren in low-income countries have shown relatively convincing cognitive effects of fish oil supplementation. The effects have been shown to be stronger in children with low socioeconomic status or malnutrition-related health problems and a low consumption of fish and very little n-3 PUFA [74,75]. However, no overall cognitive effects were found after fish oil supplementation of 6–11 year-old South African children with poor iron and n-3 LC-PUFA status [75], but paradoxically an adverse effect of fish oil was observed on memory mainly in girls and specifically those with iron deficiency anemia. Little research has been performed on the effects of n-3 LC-PUFA on brain functions in school-aged children from high-income countries. One functional magnetic resonance imaging study showed that DHA supplementation was associated with increased activation of the prefrontal cortex and better reaction time during sustained attention in healthy 8–10 year-old boys [76]. In a cross-over intervention trial with more than 800 schoolchildren we have recently found that healthy school meals rich in fish improve school performance [77]. Some observational studies have also observed a positive association between n-3 LC-PUFA intake and cognitive performance. A study of 4000 American children found that the association between n-3 LC-PUFA intake and cognitive performance was stronger in girls than in boys [78]—again, an example of gender-related nutrition. Four randomized trials have supplied schoolchildren from high-income countries specifically with n-3 LC-PUFA [79,80,81,82]. Three of these studies found some beneficial effects on cognition or school performance of 0.4–1 g/day of n-3 LC-PUFA, while the study that did not find any effect supplied only around 0.2 g/day [79]. This dose-response effect is however not always consistent, as a three-armed study in 90 British 10–12 year-old children found a beneficial effect of 0.4 g/day of DHA on word recognition, but poorer performance in children who had 1 g/day [80].

In the last mentioned study, all the DHA supplemented children had a more relaxed mood compared to controls [80], which is consistent with another trial that found an apparent effect on mood, *i.e.*, a reduction in impulsivity and anti-social behavior, in 450 healthy 8–10 year-old children supplemented with fish oil *versus* olive oil [81]. Similar behavioral effects were also observed in one of the South African studies, which showed a decrease in physical activity during school hours, less oppositional behavior, inattention and lower scores on a rating scale of traits of attention-deficit hyperactivity disorder (ADHD) after fish oil supplementation [82]. Comparable behavioral effects have also been indicated, although not firmly proven, in children with ADHD [83] and in addition, one study has found an association between *FADS* SNPs and the development of ADHD, specifically in the context of prenatal alcohol exposure [84]. It is difficult to draw any firm conclusions based on the results of these trials and observational studies in schoolchildren due to differences in dose, duration and most of all the tested outcomes. Furthermore, the studies on behavioral conditions may be biased due to methodological flaws such as limited sample size and the large number of neurological tests that were performed in most of the studies (out of which only a few showed significant effects). More well-conducted studies, adjusted for multiple test administrations, are therefore needed in order to provide more convincing evidence for an effect of n-3 LC-PUFA intake on cognitive, behavioral and emotional effects in children.

So far, the effect of gender has not been given much attention in intervention trials with n-3 LC-PUFA in preschool and school-aged children. However, as was the case in the studies on the effects of DHA in the perinatal period, a gender-treatment effect has been observed on mean arterial blood pressure after fish oil supplementation from 9 to 18 month of age in healthy Danish infants, which just as in the previously mentioned maternal fish oil supplementation study was mostly affected in boys [85]. In this case, blood pressure was reduced in the boys, which however was still counteracting the observed gender difference in the control group, resulting in an almost similar mean arterial blood pressure in the girls and boys of the fish oil supplemented group [85]. A similar gender-equalizing effect was observed on the systolic blood pressure later in infancy in a study that compared fish oil *versus* no supplement during the complementary feeding period [86]. In that study, the observed changes in systolic blood pressure were found to correlate with the previously mentioned changes in free play attention [73], which could indicate a common emotional component. Furthermore, a recent randomized controlled cross-over trial in young adults also observed a gender-specific effect of fish oil supplementation on the sensation of appetite that abolished gender differences observed after a three week intervention in the soy oil control period [87].

## 7. Neurobehavioural Outcomes beyond Childhood

Only a few studies have examined if fish oil supplementation can affect brain functions in healthy young adults, but some studies indicate that DHA may be important for cognition and behavior during late adulthood. DHA supplementation improved memory in healthy, young adults whose habitual diets were low in DHA, and the response was still modulated by sex [88] suggesting consistence with the effects found in late infancy with the achievement of gross motor milestones [89]. An observational study conducted in 6158 individuals of >65 years found that high fish consumption, but not dietary n-3 LC-PUFA intake, had a protective effect on cognitive decline [90,91]. A systematic review and meta-analysis from 2006 gathered all available evidence from observational, preclinical and clinical studies to assess the effects of n-3 LC-PUFA on cognitive protection [92]. Four of the trials have shown a protective effect of n-3 LC-PUFA only among those with mild cognitive impairment conditions [93]. In another trial with 485 subjects with mild memory complaints, an improvement of memory was demonstrated after 0.9 g/day of DHA for 24 weeks [94]. A recent meta-analysis of all randomized trials that have investigated the effect of fish oil on cognitive decline also indicated a potential beneficial effect, but only in trials that had supplied >1 g/day of DHA in subjects who at the beginning of the trail exhibited some signs of cognitive decline [90]. There are nine separate observational studies that have suggest a possible link between increased fish consumption and reduced risk of Alzheimer’s disease [95,96]. Furthermore, analysis of human cadaver brains has shown that people with Alzheimer disease have less DHA in their frontal lobe and hippocampus compared with unaffected individuals [97]. In addition, studies in mice provide support for the protective role of n-3 LC-PUFA, showing that a dietary intake of DHA induces an increase in DHA levels in the hippocampus with subsequent improvement of memory performances [98].

DHA has also been suggested to be effective in major psychiatric disorders. Most of the conducted studies have used n-3 LC-PUFA levels in RBC membranes as a measure of exposure, due to the objectivity of this measure and their high correlation with habitual dietary intake [99] in addition to their presumed reflection of brain LC-PUFA levels. Such studies have shown a significant correlation between DHA deficits and schizophrenia [100]. Life style in schizophrenia is characterized by heavy smoking, drinking, high-caloric diets, low physical activity and use of drugs that cause oxidative stress in the body. However, a recent study found no reduction of either DHA or AA in large groups of un-medicated Indian and Malaysian patients suffering from schizophrenia [101]. There is a tendency for RBC membrane levels of DHA and AA to diminish during storage, and this may happen abnormally rapidly in schizophrenia [102], possibly because of an increased oxidative stress [103]. Interestingly, several studies reported a better outcome in psychotic patients supplemented with n-3 LC-PUFA, either EPA or DHA [104].

Accumulating evidence also suggests that n-3 LC-PUFA supplementation may be efficacious for the treatment of positive and negative symptoms in patients with schizophrenia or at ultra-high risk for psychosis [105]. There is also some evidence that n-3 LC-PUFA may be relevant in relation to the pathophysiology of depression [106]. Cross-national studies indicate that higher intake of fish/seafood is correlated with lower lifetime prevalence rates of unipolar and bipolar depression [107]. In fact, depression may present with an increased production of pro-inflammatory cytokines and elevations in plasma homocysteine levels [108], and n-3 LC-PUFA have in randomized controlled trials been shown to be able to reduce both [109]. Thus, it can be speculated that n-3 PUFAs produce a positive effect on mood, partly because of the high brain content of DHA and its involvement in neurogenesis and neuroplasticity and partly due to their anti-inflammatory properties [110] as well as their effect on carbon metabolism, which is known to be of importance in relation to the metabolism of mono-aminergic neurotransmitters [111]. Some epidemiological studies have in the same way found that lower n-3 LC-PUFA intake is linked to an increased risk for emerging depressive symptoms [112]. Therefore, higher habitual dietary n-3 LC-PUFA intake may be protective against mood swings or even ultimately prevent mood dysregulation [113]. There is however a need for large well-performed randomized controlled trials in this area in order to confirm such effects.

## 8. Conclusions

The effects of DHA on brain and cognitive development have been extensively investigated in the last years. Its functional effects have been progressively, but not entirely, separated from those of AA. Clinical trials on maternal and infant dietary intakes are not entirely clear and consistent, but seem to indicate a complex interaction between the genotype pattern of *FADS*, gender, dietary intakes and lifestyle. For these reasons it is difficult to disentangle the effects of dietary DHA from the results of the randomized supplementation trials. In future studies an appropriate sample size should be calculated in order to adjust for the different variables. Mendelian trials provide a new tool to investigate the effects of LC-PUFA on cognitive development, but the interpretation of results from such trials requires an improved understanding of the biochemical effects of individual *FADS* SNPs and also needs to consider the potential differences between boys and girls.

Thus, our two questions, (1) Does the high rate of DHA accumulation in the brain have any functional importance? (2) If yes, is the endogenous synthesis of DHA high enough to support optimal functional levels of DHA in the brain? Regarding the first, there is clear evidence that DHA contributes to the visual development of infants, as also concluded by EFSA [53], but the associations with cognitive development are still not clearly interpreted [114,115], and one of the main problems could be that the effects differ between boys and girls, which needs to be considered in future trials. Due to the proposed early window of vulnerability, so far few studies have focused on the potential effects of n-3 LC-PUFA intake on cognitive and behavioral outcomes in children and young adults, but available studies indicate that the hypothesis might be worth challenging. Finally, there is preliminary evidence that DHA may ameliorate cognitive decline and affect behavioral symptoms in major neuropsychiatric disorders such as dementia, schizophrenia and depression. There is an extremely poor availability of trials on the effect of DHA supplementations that have investigated the changes in the fatty acid status as a function of the *FADS* polymorphisms. Most evidence indicates that the DHA accumulation is mainly affected by dietary intake, specifically of preformed DHA. However new studies indicate that the genetic make-up in the *FADS* gene cluster may contribute substantially to the current understanding, but that the effects may be SNP-specific and may even vary with age, or at least are most evident in the perinatal period, where the endogenous synthesis of LC-PUFA is upregulated and diet may be more easily controlled for, especially during lactation (or formula feeding).

## Abbreviations

AA: Arachidonic acid;

ADHD: Attention-Deficit Hyperactivity Disorder;

ALA: α-linolenic acid;

DHA: Docosahexaenoic acid;

EPA: Eicosapentaenoic acid;

FA%: % of the fatty acids;

*FADS*: Fatty acid desaturase gene;

LA: Linoleic acid;

LC-PUFA: Long chain PUFA;

PUFA: Polyunsaturated fatty acid;

RBC: Erythrocyte;

SNP: Single nucleotide polymorphism
